# The Need to Control Thoughts in Eating Disorder Outpatients: A Longitudinal Study on Its Modification and Association with Eating Disorder Symptom Improvement

**DOI:** 10.3390/jcm11082205

**Published:** 2022-04-14

**Authors:** Lucia Tecuta, Romana Schumann, Donatella Ballardini, Elena Tomba

**Affiliations:** 1 Department of Psychology, University of Bologna, 40127 Bologna, Italy; lucia.tecuta2@unibo.it; 2 Eating Disorder Clinic “Centro Gruber”, 40125 Bologna, Italy; r.schumann.gruber@gmail.com (R.S.); donatella.ballardini@yahoo.com (D.B.)

**Keywords:** metacognitions, metacognitive beliefs, eating disorders, cognitive behavioral therapy

## Abstract

The metacognition of needing to control thoughts has been implicated in eating disorders (EDs)—specifically, in association with the drive for thinness and over-control. To date, it has yet to be investigated longitudinally in ED outpatients undergoing CBT-based treatment. The current study aims to examine whether endorsing a need to control thoughts undergoes modifications during CBT-based treatment for EDs and whether its modification correlates with treatment response in terms of reduced ED symptomatology. Seventy female ED outpatients (34 with AN, 29 with BN, 7 with OSFED) were assessed at baseline and at the end of treatment with the Metacognitions Questionnaire (MCQ), the Eating Attitudes Test (EAT-40), and the General Health Questionnaire (GHQ). Post-treatment, significant reductions were observed in MCQ-need to control thoughts. Using hierarchical linear regression analyses such decreases significantly explained the variance in observed reductions in EAT-oral control and to a lesser extent, reductions in EAT-bulimia and food preoccupation and EAT-dieting. These results underscore the importance of metacognitive change in EDs and the potential utility of CBT-based treatment in its modification. Improving ED outcomes may warrant broadening the therapeutic target of over-control and a sense of loss of control beyond dysfunctional eating behaviors to include maladaptive metacognitions that concern the need to control thoughts.

## 1. Introduction

Metacognitions refer to beliefs about thinking and concerns the awareness, understanding, and interpretation of one’s own thought processes. These beliefs are central targets in metacognitive therapy (MCT), a third-wave cognitive therapeutic approach [1]. Such beliefs have been found to be associated with higher psychopathology and negative emotions [2] and have been implicated in several psychiatric disorders, including generalized anxiety disorder [3], obsessive-compulsive disorder and panic disorder [4], depression [5], and post-traumatic stress [6].

In recent research, the exploration of metacognitions in psychiatric populations has expanded to include eating disorders (EDs) [7], and several studies have reported significant associations between metacognitions and ED symptomatology and severity [8,9,10,11]. The metacognition of needing to control thoughts has been found to be especially relevant in this clinical population. This belief represents a cognitive attempt to suppress thoughts that are deemed dangerous or worrisome in order to prevent negative outcomes and is thought to contribute to feelings of responsibility for controlling said thoughts [1]. In EDs, it has been found to correlate significantly with general ED-related psychological maladjustment [9] and to predict ED symptomatology [8]. Moreover, the need to control thought, in patients with typical anorexia nervosa was reported to predict the drive for thinness [12].

More recently, authors [13] have supported the mediating role of metacognitions in the relationship between dysfunctional beliefs, including catastrophizing, negative evaluations of the self, low frustration tolerance, and rigid demands and ED symptomatology. The metacognition of needing to control thoughts has emerged as the most important mediator of ED symptomatology and has been found to completely mediate the relationship between irrational beliefs and ED symptom severity. This indicates that the modification of dysfunctional cognitions (common targets of cognitive behavioral therapy or CBT), might be strongly associated with the modification of the metacognition of needing to control thoughts.

Taken as a whole, the literature on metacognitions in EDs [7] supports the need to investigate a possible longitudinal relationship between metacognitions and ED symptomatology. The longitudinal relationship between the attempt to control and suppress thoughts and ED symptomatology in anorexia nervosa (AN), bulimia nervosa (BN), and otherwise-specified feeding or eating disorder (OSFED) groups should be further explored, considering the correspondence found between the restrictive and control-related aspects of EDs [9,12], anorexia nervosa, and worse metacognitive profiles [7]. Moreover, the longitudinal exploration of the potential role of metacognitions in symptom reduction has important implications for CBT outcomes in EDs. Despite CBT being considered a first-line treatment for EDs (in particular, the increasingly supported evidence-based CBT-Enhanced (CBT-E) treatment model [14,15]), there is considerable room for improvement in CBT treatment retention rates and outcomes in this clinical population [16]. Various authors have called for further research on identifying which specific cognitive targets may contribute to outcomes and impact treatment response [17,18]. Accordingly, identifying additional implicated cognitive aspects such as metacognitions may lead to novel targets of therapy, to be considered among the maintenance factors already identified by the CBT-E model. However, to date, no study has investigated metacognitions over time and the possible association with symptom change in a sample of ED patients.

Therefore, this study aims to investigate the metacognition concerning the need to control thoughts longitudinally in EDs—specifically, to examine whether it undergoes modification between baseline and post-treatment in a CBT-based treatment for EDs, and whether such modifications predict changes in ED symptomatology by the end of treatment. We hypothesize that the metacognition of needing to control thoughts will undergo significant reductions. Moreover, we hypothesize that reductions in the need to control thoughts will be significantly associated with ED symptom reduction, especially ED symptomatology concerning control and restraint.

## 2. Materials and Methods

The project was approved by the University of Bologna Bioethics Committee and the Department of Psychology Ethics Committee. Informed consent was obtained from all individual participants included in the study.

### 2.1. Participants

Consecutively recruited patients (*n* = 72) who met the Diagnostic and Statistical Manual of Mental Disorders (DSM-5) criteria for the EDs [19] AN, BN, and OSFED were recruited from a specialized outpatient ED treatment center in Italy before commencing CBT-based treatment. ED diagnoses were established at intake by the consensus of a psychiatrist and a clinical psychologist independently using the Structured Clinical Interview for the DSM-5 [20]. Except for two patients who refused to participate, all invited patients took part in the study (*n* = 70). The inclusion criteria were: (a) 18 to 65 years of age; (b) with a diagnosis of AN, BN, or OSFED; and (c) within one month of beginning treatment. The exclusion criteria were: (a) a lack of capacity to consent for research; (b) ED diagnosis secondary to a physical health or metabolic condition; or (c) comorbid drug/alcohol abuse, psychotic or neurocognitive disorders, acute suicidality, or pregnancy.

### 2.2. Measures

Patients were assessed at the baseline before commencing treatment (T0) and at the end of treatment (T1) through the following self-rated questionnaires:

(1) The Metacognitions Questionnaire [21] is a self-report questionnaire with 65 Likert scale items assessing five positive and negative evaluations of one’s cognitive processes: positive beliefs about worry, beliefs about the need to control thoughts, cognitive confidence, negative beliefs about the uncontrollability and danger of thoughts, and cognitive self-consciousness. The Italian translation (M. Brazzelli and G. Cocchini) of the MCQ-65 provided in Wells’ [22] treatment manual for anxiety disorders was used. In the current study, only the need-to-control-thoughts scale, composed of 16 items, was used. Cronbach’s alpha was 0.86 for beliefs about the need to control thoughts, in line with the validation of the original English version [1].
(2) The General Health Questionnaire 30-item version [23] is an instrument used for evaluating depressive and anxiety symptoms, sleeping problems, social functioning, well-being, and coping abilities. A composite global score is used. Higher scores reflect a greater impairment of mental health. The GHQ global score was developed as a screening measure to detect cases that are likely to have or be at major risk of developing psychiatric disorders. Cronbach’s alpha coefficients for the GHQ-30 have been tested in various empirical studies in community samples, ranging from approximately 0.82 to 0.93. Test–retest reliability coefficients varied from 0.50 to 0.90, whereas validity correlations with outcome scores from psychiatric structured interviews ranged between 0.65 and 0.70. In this study, the Italian version was applied [24].
(3) The Eating Attitudes Test-40 [25] is a 40-item Likert scale screening measure used to identify behaviors and cognitive patterns associated with EDs, where a greater total score indicates a higher ED severity. The measure yields a total score and three subscale scores: dieting, bulimia and food preoccupations, and oral control. The dieting subscale concerns a preoccupation with being thinner and a tendency to avoid high-calorie food. Bulimia and food preoccupations relate to the items that reflect thoughts about food, while the oral control subscale describes attempts to control eating, and the perceived social pressure to gain weight. The measure shows excellent psychometric properties [25]. In this study, we used the Italian version of the EAT-40, which has been validated [26] and exhibits good psychometric properties, with reported Cronbach alphas of 0.80 for the dieting subscale, 0.70 for the bulimia and food preoccupations subscale, and 0.83 for the oral control subscale.
(4) Body mass index (BMI) and illness duration in months were identified from medical records. BMI in adolescents (age < 20 years) was checked against the normative weight percentiles for the Italian population [27], with correspondence between AN diagnosis and underweight status, between both BN and OSFED diagnoses, and against normal weight or over-weight status.

### 2.3. Treatment

Treatment consisted in CBT for EDs [28] and a nutritional rehabilitation program, as recommended by the practice guidelines for the treatment of ED patients [29]. Treatment was provided in a multidisciplinary clinical outpatient setting and consisted of individual weekly sessions composed of the traditional CBT protocol for EDs. The average duration of treatment for patients was six months. Salient elements of the treatment program included cognitive behavioral techniques, such as identifying maladaptive cognitions regarding weight, shape, and eating; cognitive restructuring; the use of self-monitoring; behavioral homework and experiments; exposure to avoided foods and nutritional rehabilitation elements; focusing on normalizing eating and weight gain and restoration; psycho-education on nutrition, weight restoration, and health consequences of the illness; and support to enhance therapeutic adherence. Multidisciplinary integration was facilitated by daily case discussions between psychotherapists and nutritional physicians of clinically useful information to tailor sessions to emerging themes and the needs of single patients.

### 2.4. Data Analysis

Descriptive statistics were run for socio-demographic and clinical characteristics. *t*-tests for paired samples were conducted to test changes in MCQ and EAT-40 subscale scores between T0 and T1.

Subsequently, a linear regression analysis was performed to determine the contribution of changes in MCQ-need to control thoughts (ΔMCQ = MCQ at T1−MCQ at T0) to explaining the variance in the observed reductions in the EAT subscale scores. Firstly, change scores were calculated for EAT subscale scores, including EAT-oral control (ΔEAT-oral control = EAT-oral control at T1−EAT-oral control at T0), EAT-bulimia and food preoccupations (ΔEAT-bulimia and food preoccupations = EAT-bulimia and food preoccupations at T1-EAT-bulimia and food preoccupations at T0), and EAT-dieting (ΔEAT-dieting = EAT-dieting at T1–EAT-dieting at T0). Age, baseline BMI, illness duration, and GHQ baseline scores were inserted as covariates to control for both socio-demographic factors and illness severity, in terms of both BMI and illness duration, and general psychopathology levels (GHQ baseline total score). Secondly, dummy variables for the three ED diagnostic groups were created to test for any diagnostic differences in the prediction of ΔMCQ in ΔEAT subscale scores. Socio-demographic and clinical covariates were inserted in Block 1, ED diagnoses in Block 2, and ΔMCQ in Block 3.

Prior to conducing hierarchical multiple regressions, the relevant assumptions of this statistical analysis were tested. A sample size of 70 was deemed adequate. The assumption of singularity was also met as the independent variable, and covariates (ΔMCQ-Need to control thoughts, age, GHQ baseline total scores, baseline BMI, illness duration) were not a combination of other independent variables. An examination of the correlations revealed that no independent variables were highly correlated. Residual and scatter plots indicated that the assumptions of normality, linearity, and homoscedasticity were all satisfied [30]. In all analyses, the level of significance was set at *p* < 0.05 (two-sided). The Statistical Package for Social Sciences (SPSS) was used for all calculations. Cohen’s d effect sizes were calculated [31], where d > 0.80 is a large effect and d > 1.1 is a very large effect [32].

## 3. Results

### 3.1. Sample Characteristics

Seventy ED outpatients were analyzed for changes in their questionnaire scores during treatment (34 with AN, 29 with BN, and 7 with OSFED). Participants were all female with a mean age of 23.90 ± 8.66 years. The mean of illness duration was 6.66 ± 6.29 years. The mean BMI at baseline by diagnosis was 16.17 ± 1.79 kg/m^2^ for AN, 22.76 ± 4.08 kg/m^2^ for BN, and 18.99 ± 8.08 kg/m^2^ for OSFED. Most of the sample were single (87.1%), while 7.1% were married and 2.9% were separated or divorced. Most patients had a high school diploma (47.8%) and 29.9% had a middle school diploma, while 22.3% had graduated from university.

### 3.2. Paired t-Tests

By the end of treatment, the MCQ-need to control thoughts underwent significant changes. *t*-tests for paired samples were also run for EAT subscale scores. All the EAT subscale scores saw a significant reduction. See Table 1.

### 3.3. Regression Analysis

Analysis revealed that reductions in MCQ-need to control thoughts significantly explained the variance in the observed reductions in ED symptomatology in terms of all EAT subscales, including EAT-oral control, EAT-bulimia and food preoccupations, and EAT-dieting. Age, baseline BMI, illness duration, GHQ total scores at baseline, and ED diagnostic group did not significantly contribute to explaining the relationship between reductions in MCQ-need to control thoughts and reductions in ED symptomatology. See Table 2 for regression coefficients.

## 4. Discussion

To the best of our knowledge, the current study is the first to examine whether the metacognition regarding the need to control thoughts changes longitudinally in CBT-based treatment in a sample of ED outpatients. Moreover, the current study investigated whether a reduction in the endorsement of this metacognition improved treatment response in terms of reductions in ED symptomatology.

In the current sample, despite not directly targeting maladaptive cognitions, the metacognition of the need to control thoughts was significantly reduced by the end of CBT treatment. In line with this finding, a previous study on depression found that both maladaptive cognitions (representing content of thoughts) and targeted metacognitions (representing another level of cognitions), including the need to control thoughts and positive and negative metacognitions, had undergone reductions following Metacognitive Training for Depression [5], indicating that cognitively oriented therapies may change cognitive content and processes of various types and levels independently of their being directly tackled.

Using the CBT framework as a reference, it is possible to consider that metacognitions most likely improve through the cognitive restructuring of negative and maladaptive evaluations, which is a cardinal strategy of CBT [33]. Indeed, cognitions and cognitive aspects are focused upon and modified by cognitive restructuring in recommended CBT models for EDs for adults with AN, BN, and binge-eating disorder (BED) [29,34].

Qualitative themes of metacognitions which have been analyzed in EDs [35] indicate that the need to control thoughts is expressed in the endorsement of beliefs such as “all negative thoughts are bad, wrong or abnormal and need to be addressed and controlled”, revealing catastrophic interpretations and dichotomic thinking. Such patterns of thought are commonly challenged with cognitive restructuring, which patients learn to apply by themselves [33].

Moreover, according to cognitive theory, while maladaptive metacognitions conceptually differ from maladaptive cognitions, cognitive restructuring modifies dysfunctional evaluations regardless of the specificity of the content and inferences of events [36]. As authors have previously underscored, overlaps between conceptualizations of maladaptive cognitions exist across cognitive models [37], and the concept of meta-experiences regarding one’s own internal states is not new to second-wave CBT approaches. Ingram [38] underscored the importance in mental illness of self-focused attention and the awareness of self-referent, internally generated information, while Ellis [39] identified the role of meta-emotions in psychopathology, also known as the “secondary problem”, wherein patients experience distress about being distressed. Cognitive restructuring, central to CBT, is also the suggested strategy for the modification of metacognitions in metacognitive therapy [1].

The reduction in the endorsement of the maladaptive metacognition of the need to control thoughts explained the significant variance in the observed decreases in ED symptomatology. It is clinically relevant and worthy of note that changes in the need to control thoughts were more highly associated with changes in controlled and restrictive ED behaviors, primarily the oral control subscale. In line with some evidence from previous cross-sectional studies, the authors found that the need to control thoughts predicted the drive for thinness in one study [12], while in another, the metacognitive belief of needing to control thoughts was the most important predictor, among other metacognitions, of the global EDE-Q scores and EDE-Q subscales [40] concerning restraint, eating, shape, and weight concerns, after controlling for BMI and age [8].

Moreover, the metacognition of needing to control thoughts was found to significantly correlate with the over-control scale of the EDI [41] instrument [9]. Furthermore, among other metacognitions, the need to control thoughts correlated the most with all EDE-Q [40] scales, including restraint [8]. As the need to control thoughts is hypothesized to generate feelings of responsibility for controlling thoughts in order to try to avoid negative outcomes, in patients with AN and BN this might generally lead to a loss of control, over-eating, and over-managing weight. This suggests that perceiving poor control in both cognitive and behavioral terms may contribute to the motivation of being thinner [42]. On the other hand, these findings may reflect the general notion that restrictive ED aspects may be associated with a worse metacognitive profile, as patients with AN generally seem to engage in maladaptive attempts to control cognitive-affective states to a greater extent, compared to other diagnostic groups, according to a recent review of cross-sectional studies [7].

A mechanism of change that might explain the relationship between reductions in the metacognition of the need to control thoughts and reductions in ED symptoms may be hypothesized based on theoretical frameworks for EDs and theories on metacognitive processes. Clinical cognitive theories [14,43] propose that patients enact dysfunctional eating behaviors to avoid or cope with their negative emotions, and to escape the awareness of a negative or unpleasant emotional state. Engaging in attempts to control and suppress thoughts might be seen as an additional strategy to avoid negative emotions and unwanted internal states (i.e., metacognitions) that are deemed too distressing [44,45,46]. Since CBT challenges maladaptive thought patterns while explicitly identifying associated negative emotion states, the experiential avoidance of internal states and maladaptive metacognitions may be diminished [35]. In support of this notion, the presence of a high need to control thoughts was found to be associated with a lack of emotional awareness and to predict binge eating in a previous study [44].

### 4.1. Implications

Implications for clinicians concern the possible expansion of the well-known and targeted ED features of over-control and sense of loss of control, to maladaptive mental processes such as metacognitions, thus going beyond control-related aspects in dysfunctional eating behaviors. Such considerations may be applied across all EDs, excluding BED patients not included in the present study, as no significant differences between groups emerged. Indeed, qualitative data support a transdiagnostic view of metacognitions in EDs, as they find no substantial differences between diagnostic ED groups [35]. However, the current findings may be due to the small sample size used—in particular, for the OSFED group. Nonetheless, a sense of control plays a pivotal role not only in ED etiology [47], as clinical researchers have hypothesized for quite some time [28,48,49,50,51], but possibly in how ED patients relate to their overall cognitions, as others have suggested [42]. The consideration of cognitive aspects associated with general psychopathology in EDs is in line with a review of network analyses applied to EDs, where ineffectiveness, interoceptive awareness, and affective problems appeared as central symptoms, in addition to the core ED symptoms of overvaluation of body shape and weight and cognitive restraint. Consequently, general psychopathology (which includes maladaptive metacognitions) should be considered beyond ED-specific symptomatology in ED treatment [52].

CBT models and treatment protocols for EDs may benefit from considering metacognitions. More generally, they may benefit from considering the meta-experiences regarding one’s thought processes and internal states, including those surrounding attempts to control and suppress thoughts that are deemed dangerous and unwanted. This could be integrated as an additional maintenance factor that should be targeted along with others identified in the increasingly supported CBT-E model [14,15]. To date, only an integrated model of CBT and metacognitive therapy (MCT) has been proposed for bulimia nervosa [53]; however, a transdiagnostic MCT model for EDs has not yet been tested in a randomized controlled trial [35], and only one preliminary study exists that presents a case series with promising results of MCT applied to BED patients [53].

### 4.2. Limitations and Future Directions

Limitations of the current study include the small sample size and the lack of follow-up data. Moreover, the use of BMI as a measure of illness severity might be misleading for the subgroup of underage participants, for whom considering weight percentiles might have been more appropriate. While the current study supports the ability of CBT-based treatment in modifying metacognitions, future studies might provide additional information on which therapeutic ingredient acts upon metacognitions specifically. Other areas that are closely related to metacognitions still require further investigation in EDs, such as maladaptive perseverative thinking (e.g., rumination or worry), thought suppression and avoidance, and threat monitoring [1], especially considering their central role in Wells’ model of psychological disorders.

The current findings support the need to further explore metacognitive change in ED treatment response, as the results suggest that CBT-based treatments in EDs might to some extent modify metacognitions, thus contributing to improved ED symptomatology. Exploring metacognitions within treatment settings as well as their associations with ED symptom change is warranted, given less-than-optimal treatment retention rates and outcomes in EDs and the need to further identify specific cognitive targets associated with better outcomes and treatment responses [17,18]. Metacognitions might be clinically useful cognitive targets in EDs, as preliminary case series suggest [53].

## Figures and Tables

**Table 1:** Paired *t*-test for changes in MCQ and EAT subscale scores between baseline and end of treatment.

	T0 Mean ± SD	T1 Mean ± SD	T _(df)_	*p*	r(*p*)	d
MCQ-Need to control thoughts	27.84 ± 8.02	24.61 ± 6.67	3.510_(69)_	<0.001	0.464 (<0.001)	0.438
EAT-Oral control	7.94 ± 5.47	3.86 ± 4.86	6.631_(69)_	<0.001	0.507 (<0.001)	0.788
EAT-Bulimia and food preoccupations	7.87 ± 4.45	3.79 ± 4.10	7.443_(69)_	<0.001	0.426 (<0.001)	0.954
EAT-Dieting	18.39 ± 10.47	9.76 ± 9.60	6.611_(69)_	<0.001	0.425(<0.001)	0.859

Note: df, degrees of freedom; EAT, Eating Attitudes Test; MCQ, Metacognitions Questionnaire; *p*, significance; r, correlations between T0 and T1; SD, standard deviation; T0, baseline pre-treatment; T1, post-treatment.

**Table 2:** Regression analysis for changes in metacognitions predicting changes in ED symptomatology.

Model	B	95% CI for B	β	*t*(*p*)	F(*p*)	R	R^2^	ΔR^2^
Model Outcome: Δ EAT-40 Oral control								
Constant	−3.007	(−8.539, 2.526)		−1.087 (0.281)	5.141 (<0.0001)	0.609	0.371	0.242
Age	−0.071	(−0.251, 0.109)	−0.121	−0.791 (0.432)				
Baseline BMI	0.102	(−0.211, 0.416)	0.091	0.651 (0.517)				
Illness duration	−0.023	(−0.271, 0.225)	−0.028	−0.186 (0.853)				
Baseline GHQ total score	−0.083	(−0.205, 0.040)	−0.142	−1.346 (0.183)				
*AN vs. BN*	−1.865	(−1.088, 4.818)	−0.180	−1.263 (0.211)				
*AN vs. OSFED*	−2.469	(−1.295, 6.233)	−0.146	−1.312 (0.194)				
*BN vs. OSFED*	−0.604	(−4.419, 3.210)	−0.058	−3.17 (0.752)				
Δ MCQ- Need to control	0.341	(0.200, 0.482)	0.514	4.866(<0.0001)				
Model Outcome: Δ EAT-40 Bulimia and food preoccupations								
Constant	0.113	(−5.319, 5.544)		0.041 (0.967)	2.936 (0.010)	0.502	0.252	0.122
Age	−0.045	(−0.222, 0.132)	−0.085	−0.512 (0.610)				
Baseline BMI	−0.088	(−0.396, 0.219)	−0.087	−0.574 (0.568)				
Illness duration	−0.038	(−0.281, 0.206)	−0.052	−0.311 (0.757)				
Baseline GHQ total score	−0.010	(−0.130, 0.111)	−0.019	−0.161 (0.873)				
*AN vs. BN*	1.442	(−1.457, 4.341)	0.154	0.995 (0.324)				
*AN vs. OSFED*	−2.286	(−5.981, 1.409)	−0.249	−1.237 (0.221)				
*BN vs. OSFED*	−3.728	(−7.473, 0.017)	−0.399	−1.991 (0.051)				
Δ MCQ- Need to control	0.218	(0.073, 0.353)	0.365	3.153 (0.002)				
Model Outcome: Δ EAT-40 Dieting								
Constant	−9.723	(−22.046, 2.600)		−1.578 (0.120)	3.537 (0.003)	0.537	0.289	0.189
Age	−0.053	(−0.454, 0.348)	−0.043	−0.265 (0.792)				
Baseline BMI	0.378	(−0.321, 1.076)	0.160	1.081 (0.284)				
Illness duration	−0.144	(−0.697, 0.408)	−0.085	−0.523 (0.603)				
Baseline GHQ total score	−0.125	(−0.398, 0.148)	−0.103	−0.915 (0.364)				
AN vs. BN	2.025	(−8.601, 4.552)	0.093	0.616 (0.540)				
AN vs. OSFED	−6.715	(−1.669, 15.099)	−0.190	−1.602 (0.114)				
BN vs. OSFED	−7.063	(−16.106, 1.980)	−3.26	−1.562 (0.123)				
Δ MCQ- Need to control	0.632	(0.318, 0.945)	0.455	4.031 (0.023)				

Note: AN, anorexia nervosa; BN, bulimia nervosa; OSFED, otherwise specified feeding or eating disorder; *t*, Student’s *t*-test; R^2^, coefficient of determination; ΔR^2^ change in coefficient of determination; F, F test; EAT-40, Eating Attitudes Test 40; MCQ, Metacognitions Questionnaire.

## Data Availability

Due to the nature of this research, participants of this study did not agree for their data to be shared publicly, so supporting data are not available.
